# An application of nowcasting methods: Cases of norovirus during the winter 2023/2024 in England

**DOI:** 10.1371/journal.pcbi.1012849

**Published:** 2025-02-21

**Authors:** Jonathon Mellor, Maria L. Tang, Emilie Finch, Rachel Christie, Oliver Polhill, Christopher E. Overton, Ann Hoban, Amy Douglas, Sarah R. Deeny, Thomas Ward

**Affiliations:** 1 Chief Data Officer Group, UK Health Security Agency, London, United Kingdom; 2 Centre for Mathematical Modelling of Infectious Diseases, London School of Hygiene and Tropical Medicine, London, United Kingdom; 3 Department of Mathematical Sciences, University of Liverpool, Liverpool, United Kingdom; 4 Gastrointestinal Infections, Food Safety and One Health Division, UK Health Security Agency, London, United Kingdom; National University of Singapore Public Health, SINGAPORE

## Abstract

**Background:**

Norovirus is a leading cause of acute gastroenteritis, adding to strain on healthcare systems. Diagnostic test reporting of norovirus is often delayed, resulting in incomplete data for real-time surveillance.

**Methods:**

To nowcast the real-time case burden of norovirus a generalised additive model (GAM), semi-mechanistic Bayesian joint process and delay model “epinowcast”, and Bayesian structural time series (BSTS) model including syndromic surveillance data were developed. These models were evaluated over weekly nowcasts using a probabilistic scoring framework.

**Results:**

Using the weighted interval score (WIS) we show a heuristic approach is outperformed by models harnessing time delay corrections, with daily mean WIS = 7.73, 3.03, 2.29 for the baseline, “epinowcast”, and GAM, respectively. Forecasting approaches were reliable in the event of temporally changing reporting values, with WIS = 4.57 for the BSTS model. However, the syndromic surveillance (111 online pathways) did not improve the BSTS model, WIS = 10.28, potentially indicating poor correspondence between surveillance indicators.

**Interpretation:**

Analysis of surveillance data enhanced by nowcasting delayed reporting improves understanding over simple model assumptions, important for real-time decision making. The modelling approach needs to be informed by the patterns of the reporting delay and can have large impacts on operational performance and insights produced.

## Author summary

Norovirus is an important pathogen for infectious disease surveillance as it causes hospital strain. However, reporting delays, the time from taking a test to the data being reported to national surveillance, for norovirus cases make it challenging to understand trends in real-time This is because data in the most recent week is partially missing. The most recent cases can be estimated using methods called “nowcasting”. In this work we explore a range of different methods for nowcasting norovirus cases in England over winter 2023/24. We show that while there are differences between each model, the best performing models are those that use the partially reported data. The research shows that norovirus cases could be predicted well in real-time if an appropriate method is chosen. Additionally, we explored whether using other data, namely an online health-guidance surveillance system (NHS 111 online), can help improve model performance, but for this use case there did not appear to be a benefit.

## Introduction

Norovirus is a gastrointestinal RNA virus causing symptoms of nausea, vomiting and diarrhoea. Norovirus often causes outbreaks in enclosed settings [1], burdening health systems, particularly over winter [2,3]. Transmission was limited during lockdown periods of the SARS-CoV-2 pandemic response, followed by resurgent spreading when population mixing resumed to pre-pandemic levels [4]. The pathogen is constantly evolving with antigenic drift and shift [5] causing periodic strain replacement events [6,7], resulting in short-lived immunity. These events cause large outbreaks and elevated transmission, highlighting the importance of monitoring and improving the timeliness of insights for public health action.

Norovirus monitoring in England uses data from multiple national surveillance systems. These include positive laboratory reports from confirmed cases, of which a subset undergo molecular typing, and outbreak notifications [8]. There is a time delay between diagnostic test administration and reporting to the national surveillance data, partially attributable to norovirus not being a Schedule 2 notifiable causative agent in legislation [9]. Due to this lag, the national official statistics surveillance reports truncate time series by one week, removing partially complete data [8].

Norovirus is an excellent candidate for the application of nowcasting methods due to the inherent lag in case reporting as a non-priority pathogen. Research has been conducted on short term projections using statistical methods [10,11], though there is limited exploration of correcting for time delays in norovirus cases. Norovirus incidence is highly stochastic, with a partially seasonal pattern and high heterogeneity between localised outbreaks and national trends, making it challenging to predict. Building on nowcasting research applied during the SARS-CoV-2 pandemic [12,13] modelling can be explored to improve understanding of the real-time norovirus incidence.

In this paper, we explored the reporting delay for norovirus cases in England over the 2023/2024 winter. We retrospectively evaluate a range of methods for nowcasting this problem. Using different model structures, guide signals, and assumptions about data completeness we consider the trade-offs between approaches applicable to norovirus and beyond.

## Methods

### Ethics statement

UKHSA have an exemption under regulation 3 of section 251 of the National Health Service Act (2006) to allow identifiable patient information to be processed to diagnose, control, prevent, or recognise trends in, communicable diseases and other risks to public health.

### Data

#### Norovirus cases.

Individual test results were extracted from the Second Generation Surveillance Service (SGSS) database in UKHSA (UK Health Security Agency) [14] covering England. The database stores information on positive laboratory test results uploaded by frontline diagnostic laboratories, with a sampling bias towards health and social care settings. We deduplicated tests to obtain cases, keeping the first test per patient infection episode. Under the legislation positive norovirus diagnostic tests are required to be notified to the UKHSA, but not required within 7 days of testing [15]. Cases followed a day-of-week periodicity (S1 Fig).

We focused on two main time events for each case. Firstly, the specimen date *t* defines when the specimen was collected from the infected individual. Secondly, the report date $t_{r}$ defines the date the record is ingested into SGSS, notifying UKHSA national surveillance. As symptom onset dates are not reported, the specimen date is the most epidemiologically relevant event. Though impacted by time to treatment, the specimen date gave the least delayed representation of the epidemic’s progression compared with other available time events. The difference between report date and specimen date $d=t_{r}-t$ is the reporting delay.

**Fig 1:**
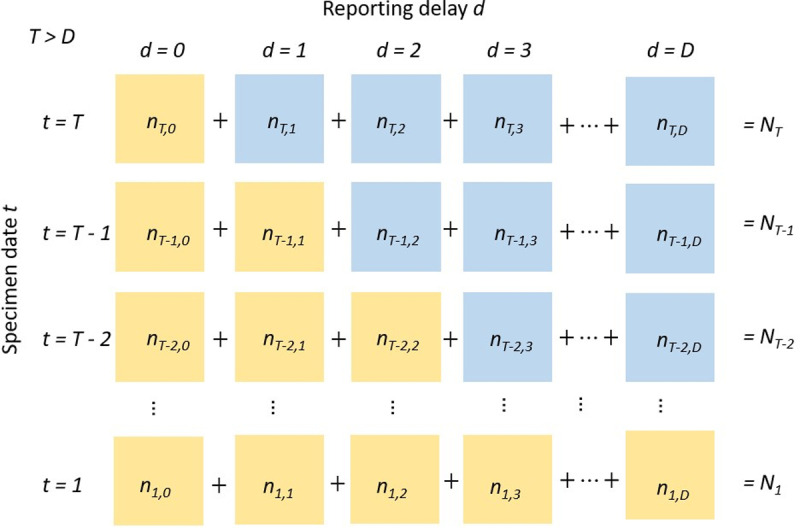
Illustration of the reporting delay structure, with elements of the 2-dimensional array. Horizontal axis represents the report delay and vertical axis the specimen date. Complete data per specimen date $N_{t}$ correspond to the sum of each row across the reporting delays. Each cell represents the case count for a given specimen date and reporting delay. Case counts are unknown in real-time when d > T-t, represented here by blue cells. The lower triangular part of the matrix, represented by the yellow cells, are the observed data, which we refer to as the reporting triangle.

To model the epidemic and corresponding delay distributions, we aggregated the data by *t* and *d* to construct a so-called data “reporting triangle” [16], illustrated in Fig 1. The nowcasts are in daily resolution, but only updated weekly (on a Sunday) to align with surveillance reporting cadence. Indexing the respective Sunday as day *T*, partial data are available up to this same day, and nowcasts need to be generated for days T,T−1,…T−6. The reporting triangle is an array with elements $n_{t,d}$, for $t\in \left\{1,..,T\right\}$ and $d\in \left\{0,\ldots ,D\right\}$, where Dis the maximum reporting delay. The element $n_{t,d}$ represents the number of case samples collected on the *t*^th^ day of the specimen date time series that were reported after *d* days. In theory, *D* could be very large. However, in practice most reporting delays are under 10 days. Therefore, for this analysis, we assume a maximum possible reported delay for final cases of 50, though each model may assume a shorter value. Final revised cases are those reported within 50 days from specimen date going forwards in this manuscript.

In real-time, cases $n_{t,d}$ cannot exist when d>T−t which introduces a right truncation. Therefore, for cases at t=T only cases with d=0 can be known, with other values 1≤d≤D unknown. The quantity of most interest used to inform decision making and proactive communications was the total cases by specimen date $N_{t}$. The reporting triangle is therefore collapsed into $N_{t}=\overset{D}{\underset{d=0}{\sum }}n_{t,d}$, the count of cases by specimen date. To support operational needs, these daily counts are also aggregated to weekly levels for ease of interpretation.

#### NHS 111 online pathways.

While there is a delay in case reporting, other data sources are complete in real-time and rapidly available, which could inform case predictions. NHS 111 Online Pathways is an algorithmic online health advice service in England used to give non-emergency guidance to individuals [17]. Users are routed to appropriate guidance given input information about their symptoms. We transformed these inputs into symptom categories, *i*, and calculate counts of symptom triages, $x_{t,i}$, by time, t, and symptom category. These counts of health seeking behaviour in the population can be used to inform case nowcasts. Symptom categories and groupings are given in S1 Table, with visualisations of the trends in S2 and S3 Figs.

### Models

The aim of our nowcasting models was to estimate the expected final number of cases identified in the most recent 7 days of the time series,$N_{t}$, given by $N_{T-6},\ldots ,N_{T}$. We take a maximum delay for final cases of 50 days to avoid including incorrectly reported historic uploads. Some models harness the partial reporting of recent cases correcting for the delay distribution, others ignore this partial reporting. We aimed to select methods that perform well against the norovirus dynamics observed. Models were tuned for appropriate hyperparameters, such as training data length, over the 4-week period using weeks ending 8 October 2023 to 29 October 2023. Using these hyperparameters the models were then sequentially refit each week up to 10 March 2024, avoiding hyperparameter selection using data later scored against. We treat the maximum reporting delay for a model as a tuneable parameter as each model handles delay distributions differently. Hyperparameters are tuned based on the average daily scores for the most recent 7 days, as outlined in the evaluation section. Model structures and assumptions are given in Table 1.

**Table 1:** Summary of key model structures, assumptions, and characteristics to compare for each model. The model runtime refers to the time taken for the nowcast model to fit, perform inference and post-processing to occur. The posterior samples are the number of samples taken from the model fit, with the burn-in referring to the number of warm up samples taken before estimating the nowcast.

Property	Baseline	BSTS	BSTS + NHS 111 online	GAM	epinowcast
Uses partial reported data	No	No	No	Yes	Yes
Parametric reporting delay distribution	–	–	–	No	Yes
Supplementary indicator signal	No	No	Yes	No	No
Bayesian	No	Yes	Yes	No	Yes
Parameter estimation method	–	Gibbs	Gibbs	REML, sampling via Metropolis-Hastings	HMC with NUTS
Model runtime	0.1s	50s	1 min 45s	10s	10 min
Posterior samples (burn-in)	–	50,000 (2,000)	50,000 (2,000)	1,000 (1,000)	1,000 (1,000)

#### Baseline.

To contextualise the performance of the models, we implement a simple baseline approach to compare against. We assumed cases predicted by day will be equal to the observed count the previous week, giving an autocorrelated prediction with day-of-week effects.

The central estimate is set as $N_{t}=\overset{T-\left(t-7\right)}{\underset{d=0}{\sum }}n_{t-7,d}$, which corresponds to the reported data from the seven days prior – matching the weekly reporting cycle in surveillance. Most norovirus cases were reported with d≤ 7 and as such this method gives predictions of near complete case numbers. We did not consider uncertainty within the baseline method. For application of the scoring methodology, prediction intervals are required. Therefore, for the baseline model the prediction intervals were assumed equal to the central estimate.

#### Generalised additive model.

We used a generalised additive model (GAM) utilising partially reported data, based on a nowcasting model for mpox [18,19]. This estimated the total number of cases with specimen date *t*, $N_{T}$, as the sum of known data that has already been reported, $n_{t,d}$, for reporting delays $d\in \left\{0,T-t\right\}$, and estimates for the unknown data yet to be reported, $n_{t,d}$ for reporting delays $d\in \left\{T-t+1,D\right\}$, i.e.,


$$N_{t}=\overset{T-t}{\underset{d=0}{\sum }}n_{t,d}+\overset{D}{\underset{d=T-t+1}{\sum }}n_{t,d}.$$ (1)

As $n_{t,d}$ $d\in \left[0,T-t\right]$ is known, $N_{t}$ has a natural lower bound of $\overset{T-t}{\underset{d=0}{\sum }}n_{t,d}$. The unknown data was modelled with a negative binomial distribution accounting for the non-negative integer values and overdispersion. Using the mean and dispersion parameterisation,


$$n_{t,d}\sim \text{NegBin}\left(\mu_{t,d},\phi \right),$$

with dispersion parameter *ϕ*. We use a log link function to model the exponential epidemic process, where $\mu_{t,d}$ depends on both *t* and *d* according to


$$\log\left(\mu_{t,d}\right)=\beta_{0}+s_{1}\left(t\right)+s_{2}\left(d\right)+\omega_{1}\left(\text{wday}\left(t\right)\right)+\omega_{2}\left(\text{wday}\left(t+d\right)\right),$$

where $\beta_{0}$ is a constant. We assumed that the number of cases vary smoothly over specimen date *t* and number of days delay *d* as $s_{1}\left(t\right)$ and $s_{2}\left(d\right)$, with random day-of-week effects $\omega_{1}\left(\text{wday}\left(t\right)\right)$ and $\omega_{2}\left(\text{wday}\left(t+d\right)\right)$ respectively. The model was fitted in *R* using the *gam* function from the *mgcv* package [20]. 1000 burn-in and posterior samples were drawn from the model using the *gratia* package [21] with a Metropolis-Hastings sampler. Samples were aggregated to $N_{t}$ (Equation 1), with prediction intervals taken using quantiles of these samples. Models were fit to the past 5 days, with cubic regression spline basis functions every l=7 days for $s_{1}\left(t\right)$ and $s_{2}\left(d\right)$, and a maximum reporting delay D=14. Model tuning is outlined in S1 Text.

#### Epinowcast.

As another nowcasting approach using partially reported data, we used a Bayesian hierarchical nowcasting framework via the *epinowcast* package [22], with the implementation described below. This approach builds on earlier nowcasting methods [23,24]. As with the “GAM” model, we have the total number of cases with specimen date *t*, $N_{t}$, the sum of known data, $n_{t,d}$ for $d\in \left\{0,T-t\right\}$, and estimates for the unknown data, $n_{t,d}$ for $d\in \left\{T-t+1,D\right\}$ (Equation 1).

Here, the reported data given the expected total number of cases, $n_{t,d}|N_{t}$, follows a multinomial distribution with a probability vector $p_{t}$ (with elements $p_{t,d}$ for $d\in \left\{0,..,D\right\}$) that is estimated jointly with the expected number of final reported cases. The expected value of final cases is taken as a generative process of the epidemic growth rate $r_{t}$, i.e.,


$$E\left[N_{t}\right]=\lambda_{t},$$

$$r_{t}=\log\left(\lambda_{t}\right)-\log\left(\lambda_{t-1}\right),$$

and $r_{t}$ is modelled by a daily random effect $\omega_{1}\left(t\right)$ and a random effect for the day of the week $\omega_{2}\left(\text{wday}\left(t\right)\right)$, to account for weekly periodicity in the underlying data,


$$r_{t}=\omega_{1}\left(t\right)+\omega_{2}\left(\text{wday}\left(t\right)\right).$$

Next, for the reporting delay model, we assume it follows the default parametric $\text{LogNormal}\left(\mu_{d},\sigma_{d}\right)$. The distribution is implemented with discretised (censored) daily probabilities and right truncation at the maximum delay *D*. The distribution is estimated with the following priors,


$$\mu_{d}\sim \text{Normal}\left(\text{log}\left(3\right),0.2\right),$$

$$\sigma_{d}\sim \text{HalfNormal}\left(0.25,0.2\right).$$

Taking *F* as the cumulative probability of $\text{LogNormal}\left(\mu_{d},\sigma_{d}\right)$, we can approximate the probability of reporting a delay of *d*, $p_{t,d}$, for d>0, as


$$p_{t,d}=\frac{F\left(d+1\right)-F\left(d-1\right)}{F\left(D+1\right)+F\left(D\right)},$$

and for d=0 as


$$p_{t,0}=\frac{F\left(1\right)}{F\left(D+1\right)+F\left(D\right)},$$

which allows us to estimate the full model. Our count per specimen date per report delay, $n_{t,d}$, is therefore parameterised by the expected final count and probability of reporting on a given day t=1,…,T,


$$n_{t,d}|\lambda_{t},p_{t,d}\sim \text{NegBin}\left({\text{λ}}_{t}\times p_{t,d},\phi \right),$$

$$N_{t}=\overset{D}{\underset{d=0}{\sum }}n_{t,d},$$

with the following overdispersion prior


$$\frac{1}{\surd \phi }\sim \text{HalfNormal}\left(0,1\right).$$

Unlike the “GAM” model, this approach introduces parametric, discrete, and truncated distributions for the reporting delay, better reflecting the reporting measurements. Models are fit in *stan* with *cmdstan* [25] using the Hamiltonian Monte Carlo (HMC) with NUTS (No-U-Turn Sampler). We ran 1000 iterations for warm-up and 1000 post-warmup iterations. A maximum reporting delay of 7 days, with a training length of 35 was selected. Model tuning and prior specification are outlined in S3 Text.

#### Bayesian structural time series.

We employed a Bayesian structural time series (BSTS) modelling approach to nowcast without harnessing partial reported case counts. The time series $N_{t}$ is truncated by 7 days, with the unknown daily counts estimated as a forecast. The BSTS allows for a state space specification with decomposition of time varying dynamics including trend, seasonality and regression effects [26]. We create two models using the *bsts* R package [27], one without regressors, the second using 111 online indicators.

The first model “BSTS” is defined by the following state space equations, where at time *t*, we have mean $\mu_{t}$, slope $\delta_{t}$ and seasonal component $\tau_{t}$, with a season as S=7 days to capture the day-of-week effects,


$$\log\left(\lambda_{t}\right)=\mu_{t}+\tau_{t}\;where\;N_{t}\sim Poisson\left(\lambda_{t}\right).$$ (2)

The equation for the mean $\mu_{t}$is given by


$$\mu_{t+1}=\mu_{t}+\delta_{t}+\eta_{0,t}\;with\;\eta_{0,t}\sim N\left(0,\sigma_{\mu }\right),$$

and the slope,


$$\delta_{t+1}=\delta_{t}+\eta_{1,t}\;and\;\eta_{1,t}\sim N\left(0,\sigma_{\delta }\right).$$

Lastly the seasonality component is determined via dummy regression variables,


$${\text{τ}}_{t+1}=-\overset{S-1}{\underset{s=1}{\sum }}{\text{τ}}_{t-s+1}+\eta_{2,t}$$

with $\eta_{2,t}\sim N\left(0,\sigma_{\tau }\right)$.

This ensures that the seasonal component ${\text{τ}}_{t}$ accounts for the cumulative seasonal effects over the specified period *S*, in our case one week. Therefore, $\log\left(\lambda_{t}\right)$ follows a local linear trend with seasonality, where the mean and slope of the trend are assumed to follow random walks. For the “BSTS” model, a training length of 60 days was chosen, with upper limits of $\text{exp}\left(\sigma_{\mu }\right)$ and $\exp\left(\sigma_{\delta }\right)$ equal to 1.1. Model tuning is outlined in S3 Text. The models were fit via Gibbs sampling MCMC, run for 50,000 iterations with 2,000 burn in.

To produce the second model “BSTS + NHS 111 online” we update the observational level (Equation 2) to include the *i* regressor symptom category scaled counts $x_{i,t}$ in$x_{t}$

$\log\left(\lambda_{t}\right)=\mu_{t}+\tau_{t}+\beta^{T}x_{t},$where$N_{t}\sim Poisson\left(\lambda_{t}\right).$

The $\beta_{i}$ values are estimated using spike and slab priors [28] centred on zero to allow for sensible variable selection. For the “BSTS + NHS 111 online” model we choose a training length of 150 days, 5 expected regression coefficients (through the spike and slab prior), and an upper limit for $\text{exp}\left(\sigma_{\mu }\right)$ of 1.01 and $\exp\left(\sigma_{\delta }\right)$ of 1.1. Model tuning analysis is given in S3 Text.

#### Weekly aggregation.

For each model, we have prediction samples $N_{t,i}$, the daily cases prediction for sample *i* at time *t*. The models are fit at a daily resolution, and to convert to weekly resolution, the prediction samples are aggregated. The weekly data are denoted by *w*, the first day of each seven-day week, giving weekly predicted case counts as $N_{w}$ and a weekly prediction sample as $N_{w,i}$, given by


$$N_{w,i}=\overset{w+6}{\underset{t=w}{\sum }}N_{t,i}$$

from which weekly summary statistics can be derived. The predictive median and prediction intervals are generated separately at daily and weekly resolutions.

#### Ensemble.

To improve predictive performance of real-time estimates, model ensembles are often used. To contextualise our nowcasts with this common practice we produce an ensemble using the “BSTS”, ”epinowcast” and “GAM” models, the best performing approaches. A parsimonious ensemble method is chosen, where we take the mean of the prediction quantiles across the three models. For model *m* taking $Q_{x,m}\left(N_{t,m}\right)$ where $Q_{x,m}$ is a function producing the *x* quantile for each model quantile desired, across *M* models,


$$Q_{x,\text{ensemble}}\left(N_{t,\text{ensemble}}\right)=\frac{1}{M}\overset{M}{\underset{m=1}{\sum }}Q_{x,m}\left(N_{t,m}\right)$$

### Models overview. Evaluation.

 To compare the different nowcasting approaches we employ multiple scoring methods in a probabilistic framework. Throughout we take the predictive median as the central estimate for the probabilistic forecast, along with 50% and 90% prediction intervals. The interval coverage is a measure of probabilistic calibration, telling us the proportion of observations that are within given prediction interval ranges – in our case 50% and 90%. Prediction coverage closer to the nominal (50% and 90%) coverage are preferred, as they reflect well calibrated predictions.

The weighted interval score (WIS) is a proper scoring rule composed of sharpness and under/overprediction, giving an overall measure of performance where low values are preferred. The predictive median, 50% and 90% intervals were used to calculate the WIS. The weighted interval skill score is calculated as ${\text{WISS}}_{\text{model}}=1-\frac{{\text{WIS}}_{\text{model}}}{{\text{WIS}}_{\text{baseline}}}$ where ${\text{WISS}}_{\text{model}}>0$ corresponds to a model better than the “baseline” model.

The bias is a score between -1 and 1 that indicates if the model tends to underpredict or overpredict by comparing observed values to the predictive median and quantiles [29]. Values closer to -1 indicate underprediction and closer to 1 correspond to overprediction, if the bias value is 0 this means that the observed value is exactly the median and the model neither underpredicts nor overpredicts. The bias penalises forecasts more where the observed data falls in quantile levels further from the median.

The mean absolute error gives the average of the absolute difference between the median prediction and the final observed cases. This mean absolute error gives a measure of performance on the scale of prediction, so we can infer on average how close the central estimate is to the observed data.

All scoring is conducted using the *scoringutils* package [30], which has supporting documentation for each metric. The estimates are scored at daily and weekly aggregations, as well as explored by nowcast horizon *h*, where h=T−t in our case is the day-of-week predicted. Since the data is uploaded weekly, the nowcast horizon *h* corresponds to a unique day-of-week where *Sunday* will be a nowcast horizon of 0 days*,* and *Monday* will have a nowcast horizon of 6 days.

The baseline model notably does not incorporate uncertainty, setting all quantiles equal to the central estimate. In this case, it will perform poorly where coverage is a component in the score. However, the mean absolute error of the median prediction is most comparable across all models as it does not incorporate uncertainty.

## Results

**Fig 2:**
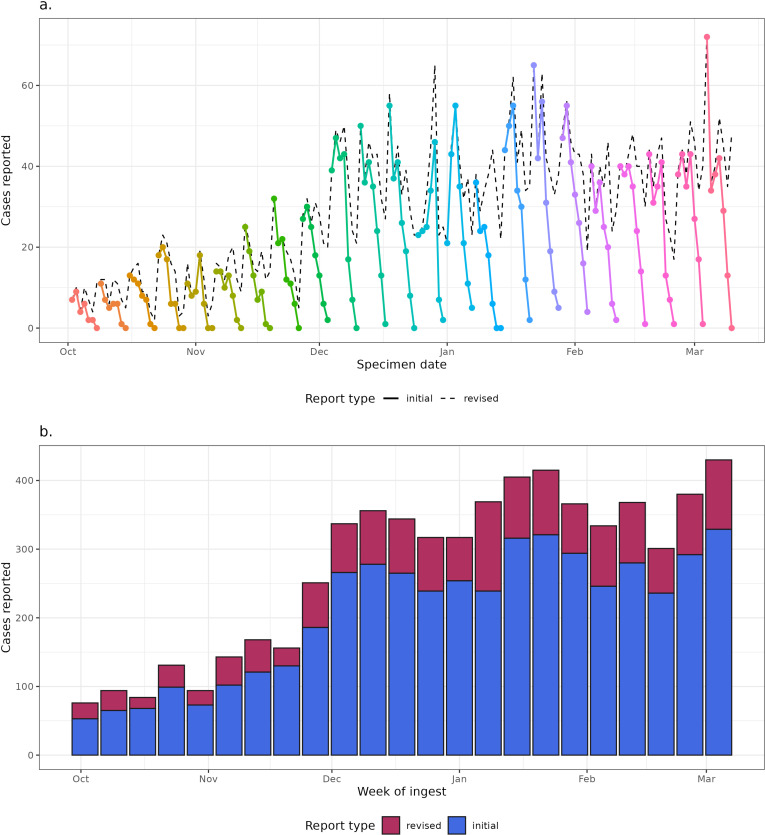
The backfilling of norovirus cases over the Winter 2023/2024 season. Without looking at the final revised cases more recent trends appear to tail off due to reporting delays. (a) daily counts of cases. The solid colour lines show the “initial” count of cases uploaded by the end of the week, the dotted black line shows the final “revised” counts uploaded after the week’s end. (b) The count of cases reported by the end of each week denoted by “initial”, with the additional cases reported after the weeks end denoted by “revised”. The end date for each week was taken as a Sunday, to produce a nowcast of data from the previous week.

**Fig 3:**
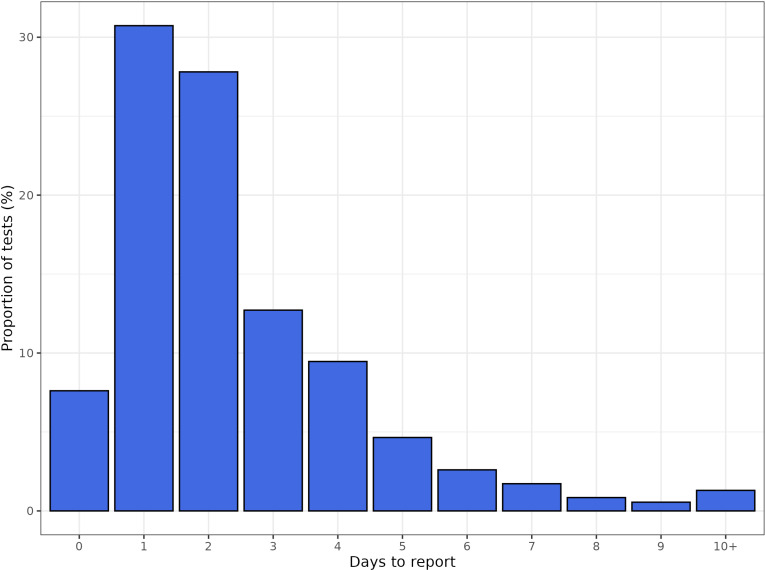
Time delay distribution of days between specimen date and report date. Includes complete data from 02-10-2023 to 10-03-2024.

Winter 2023/2024 followed the seasonal trend of increasing cases from September onwards, reaching a stable trend from December 2023 onwards. The difference between final and initial cases is largest in the most recent days each week, as expected, with $n_{t,0}$ near zero (Fig 2A). Across each week approximately 20% of the data are revisions (cases added the following week). These revisions can change the narrative of the real-time trend without correction (Fig 2B). The distribution of *d* shows few reports on d=0, a peak at 1-2 days and most reports within 7 days (Fig 3). The time varying reporting delay is given in S4 Fig, showing limited variation.

**Fig 4:**
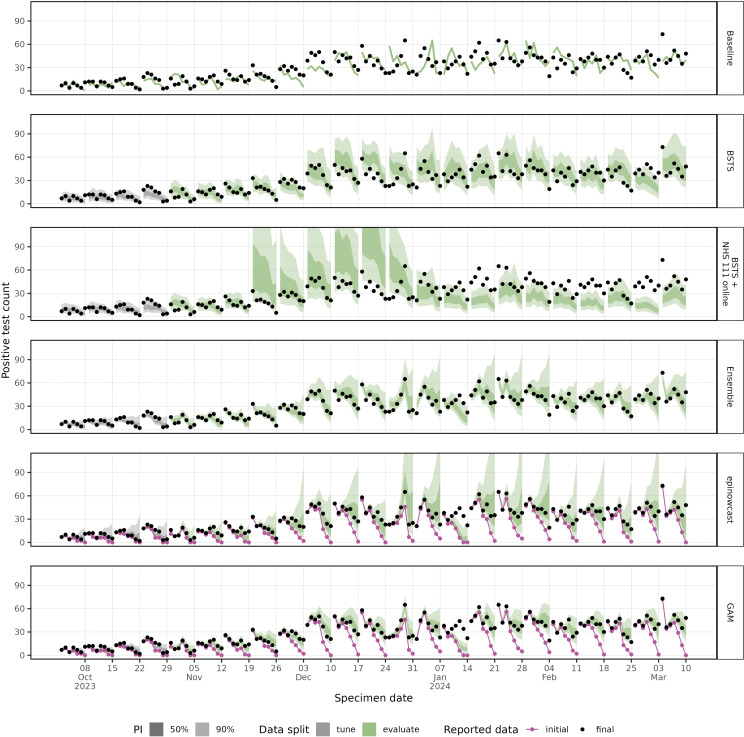
Daily predictions from all models with 50% and 90% prediction intervals against initial and final reported count of cases.

**Fig 5:**
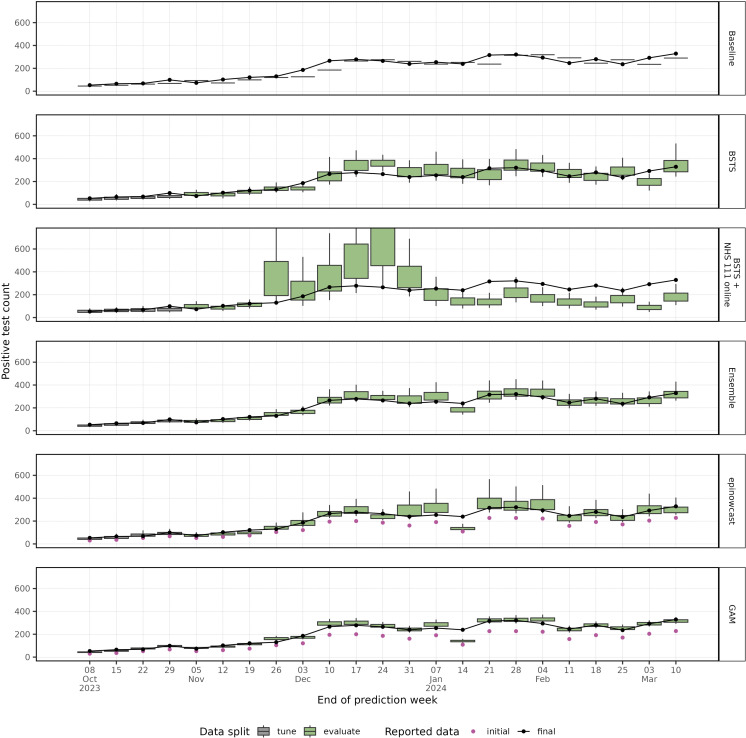
Weekly predictions from all models with 50% (box) and 90% (whiskers) prediction intervals against initial and final reported count of cases. The weekly predictions are created as the sum of sample predictions per week.

The daily and weekly nowcasts are shown over the tuning and evaluation time periods (Figs 4 and 5). Both the “GAM” and “epinowcast” models show increasing uncertainty towards the most recent date where data is more incomplete. The models using the partially complete data underpredict the complete cases in the week ending 14 January 2024, which we also see in the weekly estimates (Fig 5), though the “BSTS” is not impacted in this way. The uncertainty in the weekly estimate varies substantially by model, though the “baseline” model has no associated uncertainty. The BSTS models have wide prediction intervals compared to the “GAM”, with the “epinowcast” model prediction intervals being skewed towards higher values. As expected, the ensemble model resembles its constituent models, with wider intervals than the “GAM”, but smaller than “epinowcast”.

The overall daily and weekly evaluation scores are shown in Table 2. The “baseline” model has high WIS, expected given its small interval width. The partial reporting delay models “epinowcast” and “GAM” outperform other models across WIS and MAE. Overall, except for the “GAM”, other models underpredict (bias < 0) to varying degrees. The “BSTS” model performs better than the baseline across all daily metrics, whereas the “BSTS + NHS 111 online” performs broadly worst. Across daily and weekly scoring the “BSTS” model has the best coverage, though other models have similar values. Notably, the “GAM” and “epinowcast” models over and underpredict respectively.

**Table 2:** Breakdown of overall model scores by temporal granularity. The daily granularity shows the average daily score over the time series. The weekly granularity shows the average weekly score over the time series. The most optimal score by temporal granularity and scoring metric is in bold.

Model	Temporal granularity	WIS	Mean absolute error	Bias	50% coverage	90% coverage
Baseline	daily	7.73	7.73	−0.21	0.07	0.07
BSTS	daily	4.57	7.18	−0.07	**0.53**	**0.90**
BSTS + NHS 111 online	daily	10.28	15.36	−0.24	0.35	0.72
epinowcast	daily	3.03	4.07	−0.31	0.64	0.88
GAM	daily	**2.29**	**3.39**	**0.05**	0.57	0.89
Ensemble	daily	2.64	3.80	−0.10	0.60	0.96
Baseline	weekly	29.74	29.74	−0.39	0.00	0.00
BSTS	weekly	21.19	34.04	−0.05	**0.43**	0.83
BSTS + NHS 111 online	weekly	67.44	100.35	−0.30	0.26	0.65
epinowcast	weekly	15.61	22.35	−0.19	0.61	**0.96**
GAM	weekly	**11.56**	**16.00**	**0.03**	0.35	0.78
Ensemble	weekly	11.98	19.35	−0.08	0.35	1.00

**Fig 6:**
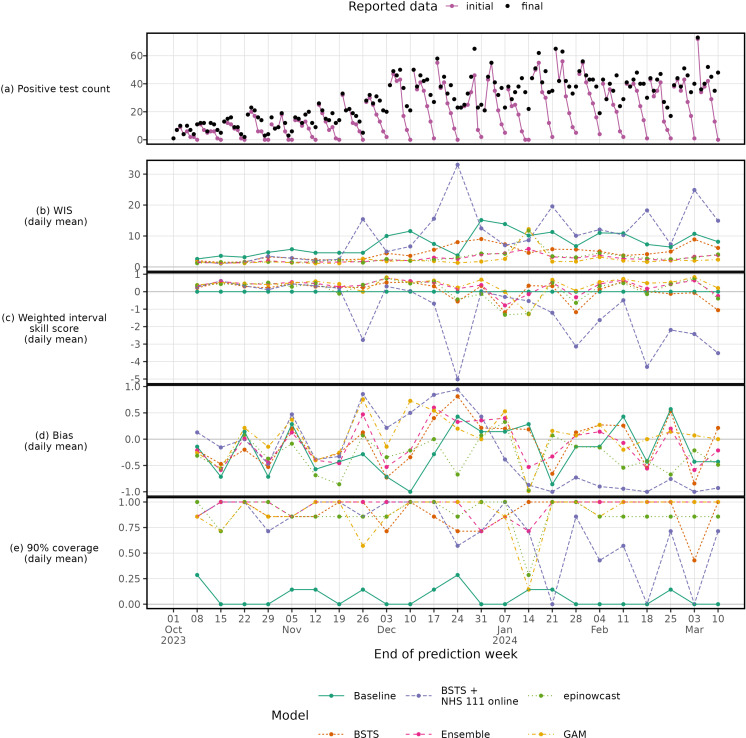
Daily count of final and initial reported cases (a) with daily mean model scores for each prediction week. The Weighted Interval Score (b), Weighted Interval Skill Score (c), Bias (d) and Coverage deviation (e) are given across models and time.

Over the evaluation period the “GAM”, “BSTS” and “epinowcast” models have improved skill over the baseline model in most but not all weeks (Fig 6C). For much of the time series, the “BSTS + NHS 111 online” model has higher WIS than the baseline model (Fig 6B). The “GAM” and “epinowcast” models have bias > 0 during the epidemic growth phase, indicating overprediction (Fig 6C). The week of 14 January 2024 the “epinowcast” and “GAM” perform markedly worse than other weeks, where initial reported data is particularly low. The “ensemble” model, as an average performed similarly to its constituent models, though this averaging helped avoid poor performance of the “epinowcast” and “GAM” models on 14 January 2024, demonstrating its utility. Further scoring at daily and weekly levels are given in S5 and S6 Figs.

**Fig 7:**
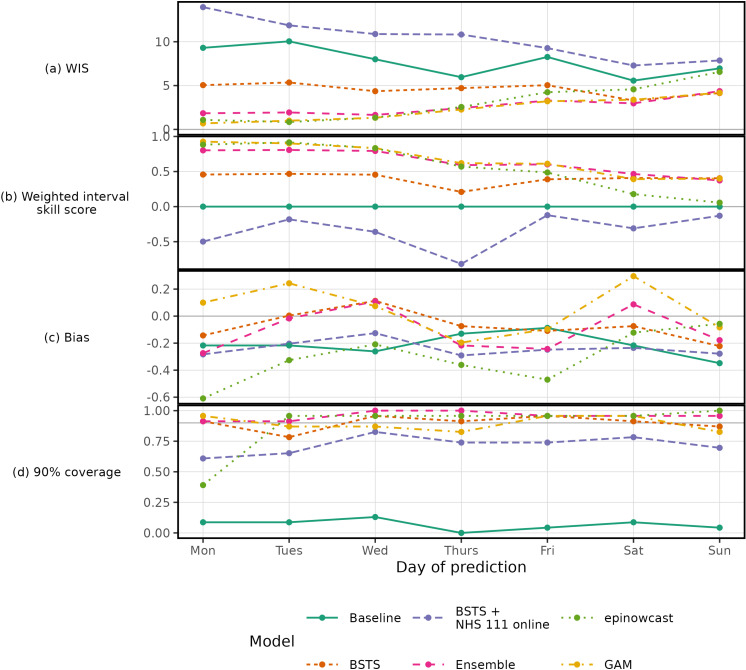
Model scores, Weighted Interval Score (WIS), weighted interval skill score, bias and coverage deviation, averaged over each day of prediction. A Monday has near complete data, whereas a Sunday has many cases not yet reported. The scores are the average over the evaluation period.

By breaking down by the day-of-week (and therefore nowcast horizon, in our case) we can explore how varying data completeness affects model performance. Relative to “baseline” the “BSTS” model exhibits a flat skill across days (Fig 7A), whereas the relative skill of the “GAM” and “epinowcast” gets deteriorates towards the end of the week (Fig 7B). The “baseline” consistently underpredicts, while “epinowcast” underpredicts at the start of the week but becomes less biased toward Sunday (Fig 7C). Compared to the “BSTS” model, the improved performance of the “GAM” model is primarily due to lower WIS early in the prediction week when data is more complete.

## Discussion

Norovirus contributes substantially to health service winter pressures through hospital outbreaks, reduced bed availability and staff absences. As such, timely surveillance is crucial for situational awareness, particularly to understand changes in the epidemic curve in the context of delayed reporting. In this work we applied a range of nowcasting approaches to norovirus cases, with the aim of understanding the current epidemic state. We have shown that harnessing partially complete data outperforms a truncate-and-forecast approach, but the performance can be sensitive to the consistency of case reporting, which is challenging in frontline health protection. The delay in reporting impacts the analysis of trends in national surveillance, so it is important official reporting exclude these partially reported days, though nowcasting can support decision making in real-time. The nowcasting problem presented is a straightforward application of time delay correction, with a small average delay, a single test type, and without considering regional or age-related variation. This may partially explain the strong performance of approximate methods in the scoring.

Nowcasting approaches are increasingly used to predict case counts by accounting for delays in reporting, and have been crucial in the recent COVID-19 pandemic and mpox outbreak [12,18,24,31]. In this analysis, we apply several modelling approaches from the epidemic literature to this problem. We compare a well-principled Bayesian implementation, “epinowcast”, which jointly models a reporting delay distribution with an underlying process model, and a more approximate but highly flexible and computationally efficient GAM-based model.

We also consider a Bayesian structural time series approach, testing the utility of incorporating leading indicators into the modelling framework. To our knowledge this is the first study to apply time delay nowcasting methods to norovirus cases, which may be more challenging to nowcast than other infectious diseases due to high levels of underreporting, regional heterogeneity and its association with outbreaks in closed settings such as care homes, schools and hospitals [32]. Despite this, several models generated operationally useful predictions of norovirus test counts, offering a substantial improvement over using truncated data (the current standard) or a naïve seasonal baseline. However, when reporting delay data is unavailable, time series forecasting presents an adaptive alternative with good coverage and performance compared to the baseline. In contrast to previous studies, we did not find including leading indicators improved our predictions [33]. This could perhaps be explained by lower signal in the indicators considered, related to confounding effects from other winter pathogens. Finally, while it has been demonstrated in a range of settings that ensembles can outperform individual models [12], in our specific context with forecast and nowcast models this was not necessarily the case.

Models incorporating reporting delays consistently performed better than forecasting approaches, showing the utility of leveraging this partial data when available. This improved performance is driven by reduced uncertainty when there is more complete reported data, early in the nowcast window. Among our models using reporting delays, we found that the time delay approximation method in the “GAM” scored slightly better than the more complex “epinowcast” model’s full joint distribution approach, in this application. The “epinowcast” has increased uncertainty due to parametrically modelling the reporting delay distribution and underlying process model. Wide intervals are penalised in scoring metrics like the WIS, however, this larger uncertainty may better reflect the uncertainty in the system. We saw that modelling based on recent distributions of reporting delays can perform poorly if these distributions change rapidly, although in these cases, the “epinowcast” model’s optional time-varying delay may be advantageous compared to a fixed distribution approach, such as the one in the “GAM”. Speed is key in a real-time modelling context, with some models being substantially faster than others, however, all approaches ran in a reasonable time (Table 1) for real-time inference. The computational expense of “epinowcast” with full Hamiltonian Monte Carlo (HMC) fitting, relative to other models, was impactful during model development. However, approximation alternatives to HMC could effectively combat this limitation.

Notably, the inclusion of 111 online pathways within the BSTS did not improve predictive performance. This could be due to multiple reasons, the community online signal may not lead cases in time, the symptom categories could be too non-specific, or the model specification could be unable to capture the relationship between signals. Further triangulation between surveillance systems could support future work in this area. Furthermore, the use of a symptom onset date may improve this modelling utility giving a better representation of the epidemic’s progression, however, given current legislation and case management practices this is unlikely to be collected in future.

The performance of some models may have been limited due to the tuning approach taken. Hyperparameter optimisation was performed on a time before the epidemic wave started, simulating a plausible real-time scenario – which may bias selection toward hyperparameters performant during flat periods of incidence. There are reporting changes in frontline healthcare delivery which can impact the performance of time delay informed models – these local practices are challenging to understand in real-time and adjust for in modelling, which should be explored further. Future work should explore how local testing practices can be incorporated into modelling directly. In addition, more exploration of historic trends could improve baseline performance, or inform modelled predictions better, though care must be taking in assuming past seasons will reflect future ones. Understanding testing pathways and real-time modelling of norovirus will be crucial for the next strain replacement event highlighting the importance of developing our understanding and preparedness.

While not a high priority pandemic potential pathogen, norovirus causes healthcare system strain and an unpleasant infection for the individual, increasing associated opportunity cost by blocking beds and elongating patient length of stay [3]. Estimating the current case burden when accounting for delayed reporting can be an important tool for supporting effective public health response. In this work we have compared the options available to correct for delayed reporting, highlighting their strengths and limitations – notably demonstrating the importance of explicitly modelling the partially complete data. This work will underpin situational awareness should the next strain replacement event occur.

## Supporting information

S1 Table The logical conditions used to define each indicator from NHS 111 pathways. The texts defining a pathway description change over time, as triage algorithms are updated by NHS 111.
(XLSX)

S1 Text Description of the GAM model tuning approach and results. (DOCX)

S2 Text Description of the epinowcast model tuning approach and results. (DOCX)

S3 Text Description of the BSTS and BSTS + NHS 111 online model tuning approaches and results. (DOCX)

S1 Fig a) the difference between each day-of-the week and the average value per week of norovirus case counts by specimen date. The figure demonstrates the periodicity of cases with lower reported values on Saturdays and Sundays. b) The autocorrelation between each day in the time series, showing how correlated each case count with lags of itself. There are notable peaks each 7 days underlining the periodicity in the time series.
(TIF)

S2 Fig The scaled values of different NHS 111 online pathway symptom trends and norovirus positive cases (black). The signals are scaled between 0-1, a.) shows the rolling 7-day mean values for indicator and case trend, where b.) shows the unsmoothed more stochastic data with day-of-week effects.
(TIF)

S3 Fig Values of each NHS 111 online pathway symptom trends. The more generic symptom categorisations such as “all pain” have larger magnitudes compared to more severe and specific symptoms such as “fever”.
(TIF)

S4 Fig The distribution of time from specimen date to report date by nowcast prediction week. The mean, median, 95% and 50% quantile intervals are given for the time delay giving a trend over time. There is a larger tail in reporting delay early in the time series, thought this is the time with fewest positive tests.
(TIF)

S5 Fig Weekly count of final and initial reported tests (top pane) with weekly model scores. (TIF)

S6 Fig Daily count of final and initial reported tests (top pane) with daily model scores. (TIF)
