# Supplementary material for: An application of nowcasting methods: Cases of norovirus during the winter 2023/2024 in England
Source: PLoS Comput Biol. 2025 Feb 21;21(2):e1012849. doi: 10.1371/journal.pcbi.1012849 (PMC11878933; doi:10.1371/journal.pcbi.1012849)
Supplement: S1 Text — (DOCX) [file pcbi.1012849.s002.docx]

**S1 Text – Generalised Additive Model**

Model structure and hyperparameter choices were made to optimise the performance of the model on the most recent 7 days of the nowcast (Supplementary Table 2). For the splines, we use cubic regression basis functions and choose the number of knots every $l$ days. Cubic regression splines were chosen as they performed (via WIS) marginally better than thin plate splines. We consider $l$ as a multiple of 7 to align with the weekly cyclicity, but also evaluate $l=3$ for reporting delay (as values for maximum delay as low as 14 days were tested) – however these models performed worse than those with a larger $l$ for the reporting delay spline. Knots every 7 days for specimen date and 7 days for reporting delay were found to be optimal, along with a training length (relating to specimen date) of 56 days and a maximum reporting delay $D$ of 14 days.


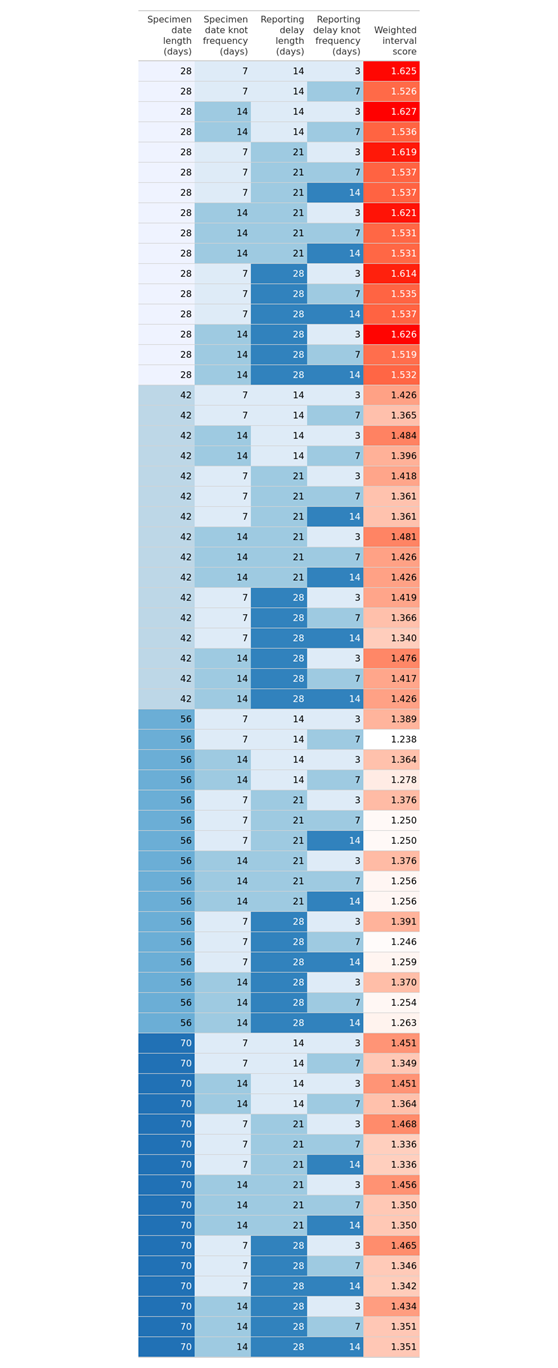


Supplementary Table 2. Average daily scores over the tuning period for the GAM model for different values of hyperparameters.
