# Supplementary material for: An application of nowcasting methods: Cases of norovirus during the winter 2023/2024 in England
Source: PLoS Comput Biol. 2025 Feb 21;21(2):e1012849. doi: 10.1371/journal.pcbi.1012849 (PMC11878933; doi:10.1371/journal.pcbi.1012849)
Supplement: S2 Text — (DOCX) [file pcbi.1012849.s003.docx]

**Text 2 - epinowcast**

We tuned two parameters: the maximum number of days to model in the delay distribution and the training length (number of days of data used to nowcast). Based on descriptive analysis of empirical reporting delays we tested maximum delays between 14 and 28 days, as well as training lengths between 21 and 49 days, shown in Supplementary Table 3. We found a maximum delay of 7 and a training length of 35 was optimum. Priors were specified to be informative, using the descriptive analysis available from the data, outlined in Supplementary Table 4. We did not tune priors to improve scoring, but rather selected sensible parameters based on known information before observing scoring results. A day of week effect for the hazard function was explored, but did not converge for all tuning results, nor did a day of week effect in the reference model converge.


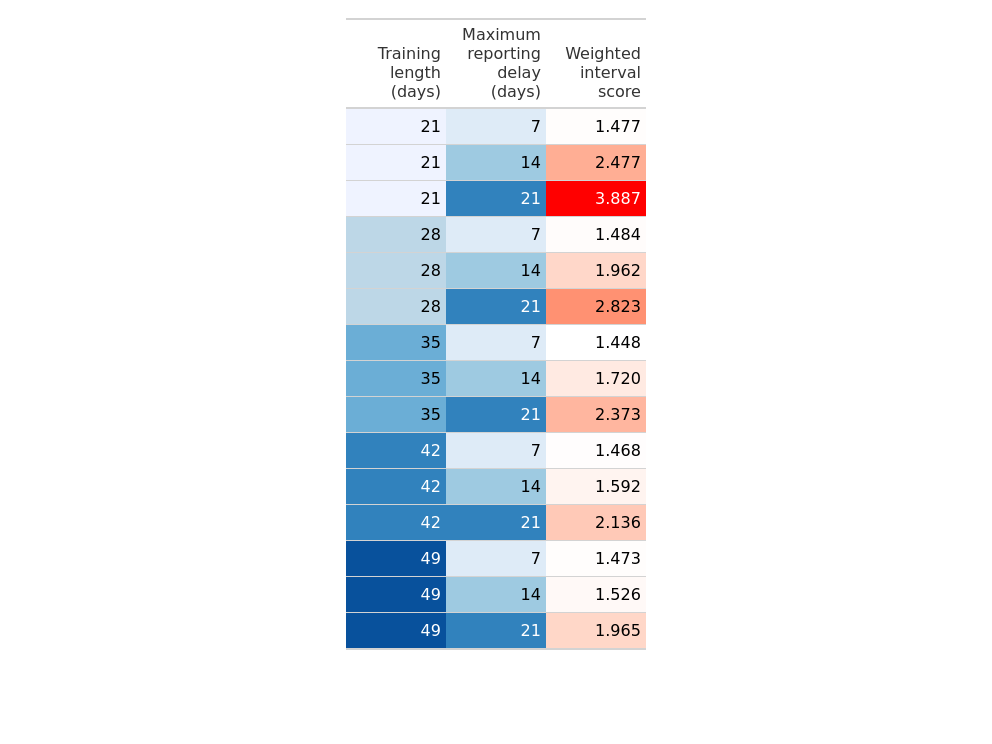


Supplementary Table 3. Average daily scores over the tuning period for the epinowcast model for different values of hyperparameters.

| **Parameter Description** | **Distribution** | **Mean** | **Standard deviation** |
| --- | --- | --- | --- |
| Log mean intercept for parametric reference date delay | Normal | log(3) | 0.2 |
| Log standard deviation for the parametric reference date delay | Zero truncated normal | 0.3 *  sqrt(log(1)-log(5)) | 0.2 |
| Standard deviation of scaled pooled parametric mean effects | Zero truncated Normal | 0.0 | 0.2 |
| Standard deviation of scaled pooled parametric standard deviation effects | Zero truncated Normal | 0.0 | 0.2 |

Supplementary Table 4. Priors specified for the epinowcast model.
