# Supplementary material for: An application of nowcasting methods: Cases of norovirus during the winter 2023/2024 in England
Source: PLoS Comput Biol. 2025 Feb 21;21(2):e1012849. doi: 10.1371/journal.pcbi.1012849 (PMC11878933; doi:10.1371/journal.pcbi.1012849)
Supplement: S3 Text — (DOCX) [file pcbi.1012849.s004.docx]

**BSTS**

We use inverse Gamma distributions for variance as priors for the standard deviations $\sigma_{\mu}$ and $\sigma_{\delta}$, specifying an upper limit for in the *sd.prior* function. The standard deviation for seasonality $\sigma_{\tau}$ is selected by the *bsts* function. Posterior samples are generated for 7 days into the future, with prediction intervals created using quantiles on the posterior samples. Hyperparameters are chosen to optimise model performance on the forecasted 7 days, based on averaged daily scores (Supplementary Table 3). We choose a training length of 60 days so that the model adapts to recent data, and an upper limit for $\exp(\sigma_{\mu})$ of 1.1 and $\exp\left( \sigma_{\delta} \right)$ of 1.1; we anticipate that the subsequent period will have greater variability compared to the tuning period and these values correspond to a 10% change in the mean and a daily accumulating 10% change in the slope respectively.

**BSTS + NHS 111 online**

For the “BSTS + NHS 111 online” model we choose a training length of 150 days, an expected model size of 5 and an upper limit for $\exp(\sigma_{\mu})$ of 1.01 and $\exp\left( \sigma_{\delta} \right)$ of 1.1, which correspond to a 1% change in the mean and a daily accumulating 10% change in the slope respectively. Scoring across each parameter are shown in Supplementary Table 4.


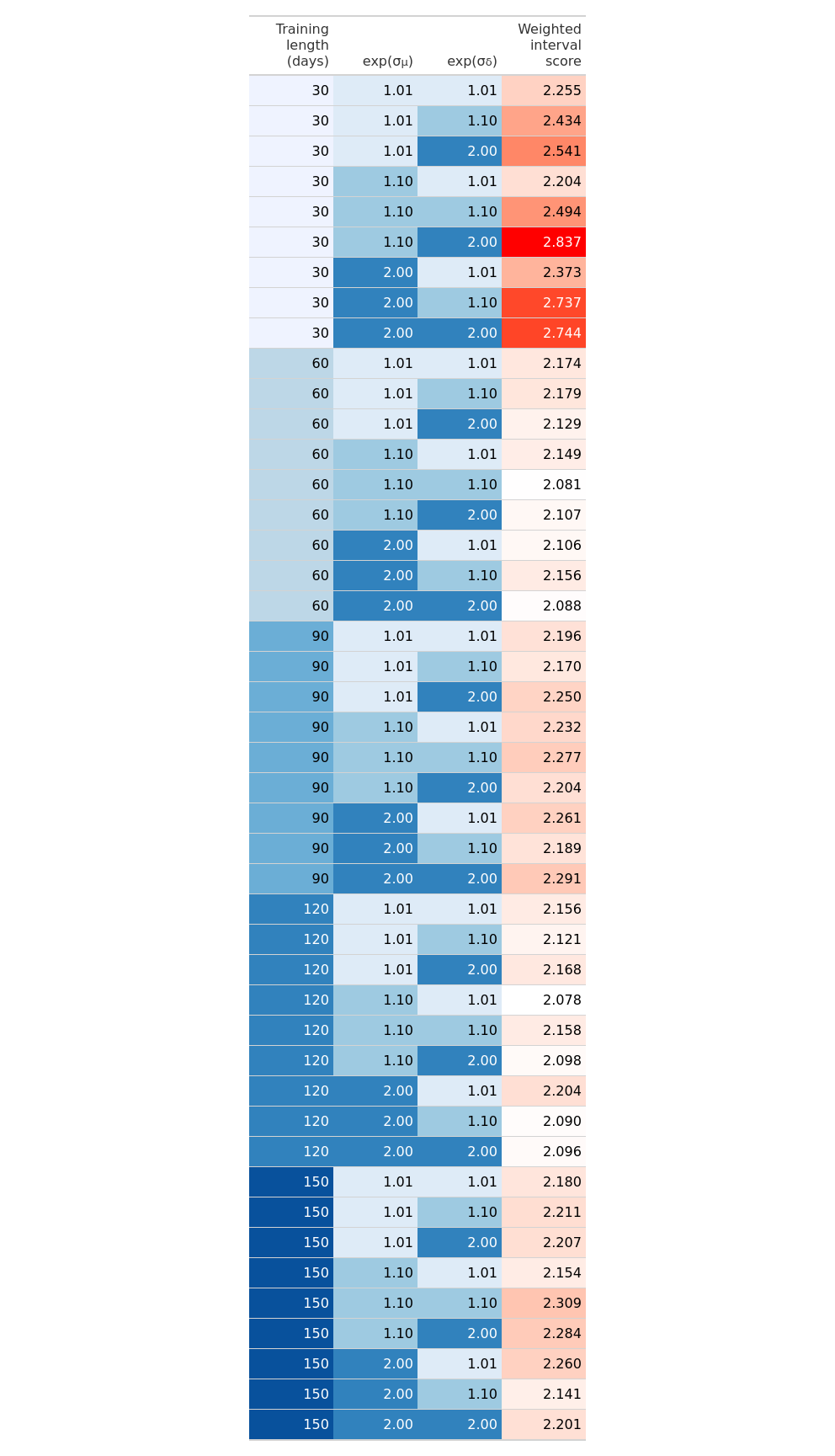


*Supplementary Table 5. Average daily scores over the tuning period for the BSTS model by training length and* $\sigma_{\delta}$ *and* $\sigma_{\mu}$ *values.*


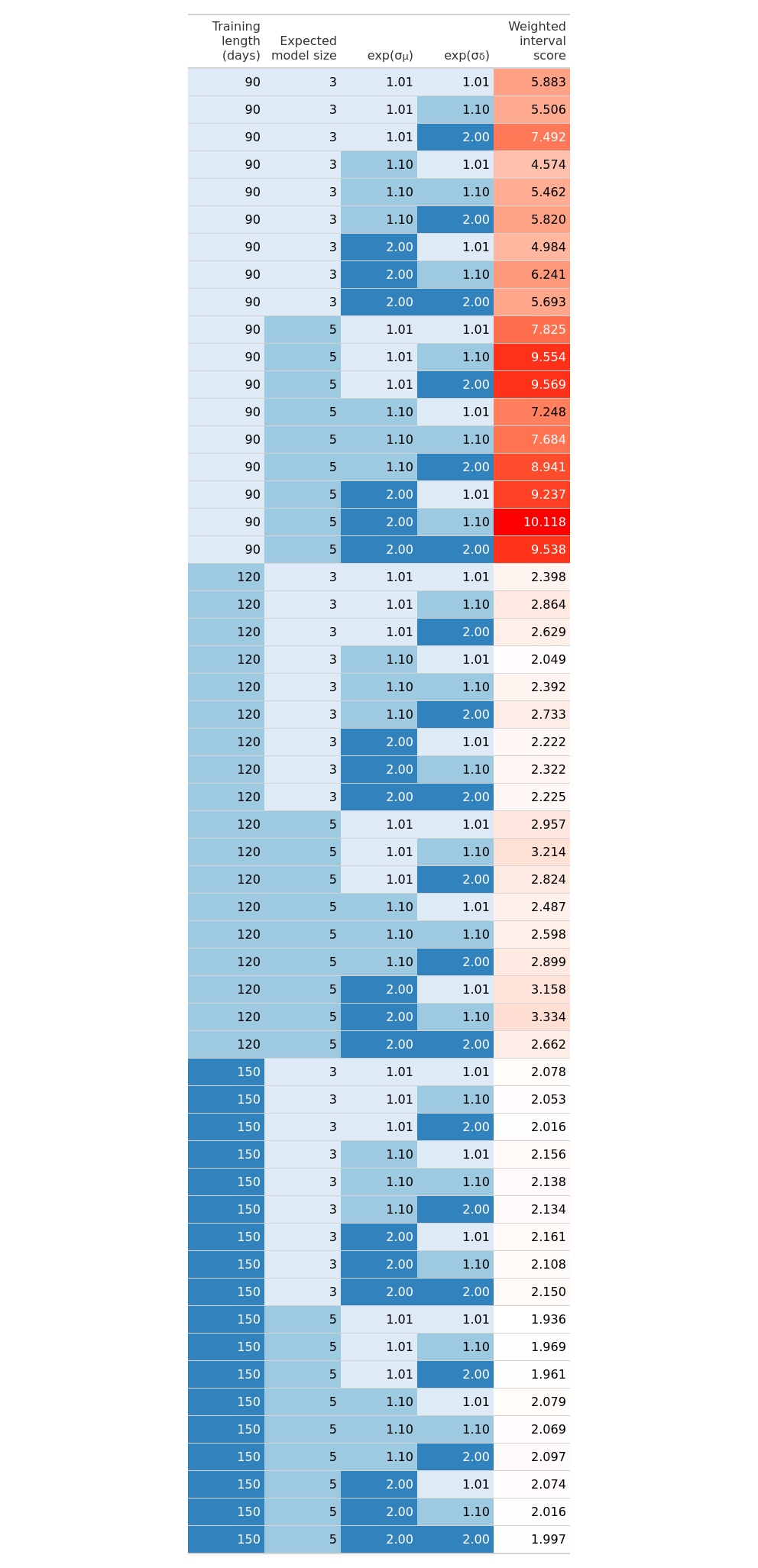


*Supplementary Table 6. Average daily scores over the tuning period for the BSTS + NHS 111 online model by training length, expected model size and* $\sigma_{\delta}$ *and* $\sigma_{\mu}$ *values.*
