# Supplementary figures and images for: An application of nowcasting methods: Cases of norovirus during the winter 2023/2024 in England
Source: PLoS Comput Biol. 2025 Feb 21;21(2):e1012849. doi: 10.1371/journal.pcbi.1012849 (PMC11878933; doi:10.1371/journal.pcbi.1012849)

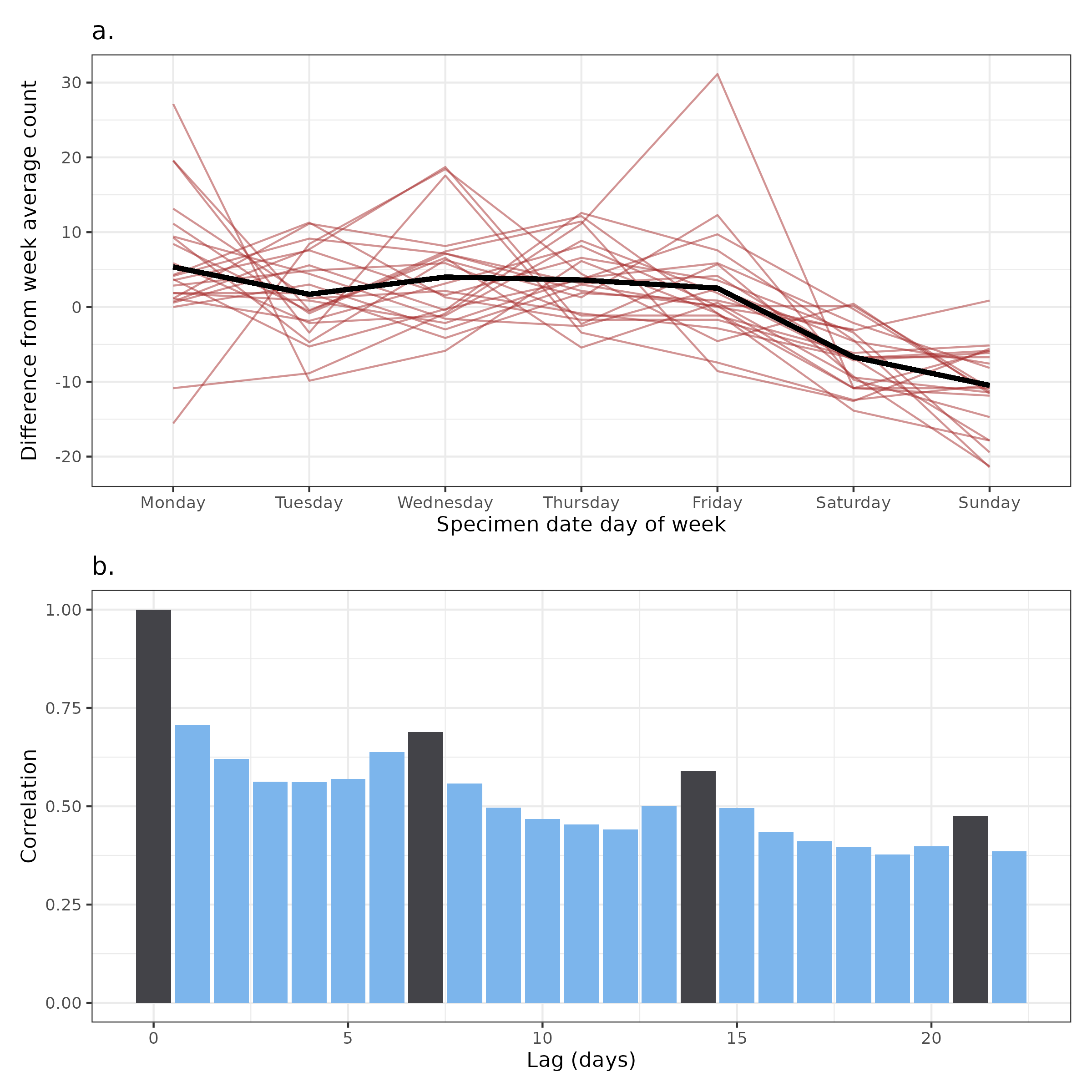

Supplement: S1 Fig — a) the difference between each day-of-the week and the average value per week of norovirus case counts by specimen date. The figure demonstrates the periodicity of cases with lower reported values on Saturdays and Sundays. b) The autocorrelation between each day in the time series, showing how correlated each case count with lags of itself. There are notable peaks each 7 days underlining the periodicity in the time series. (TIF) [file pcbi.1012849.s005.tif]

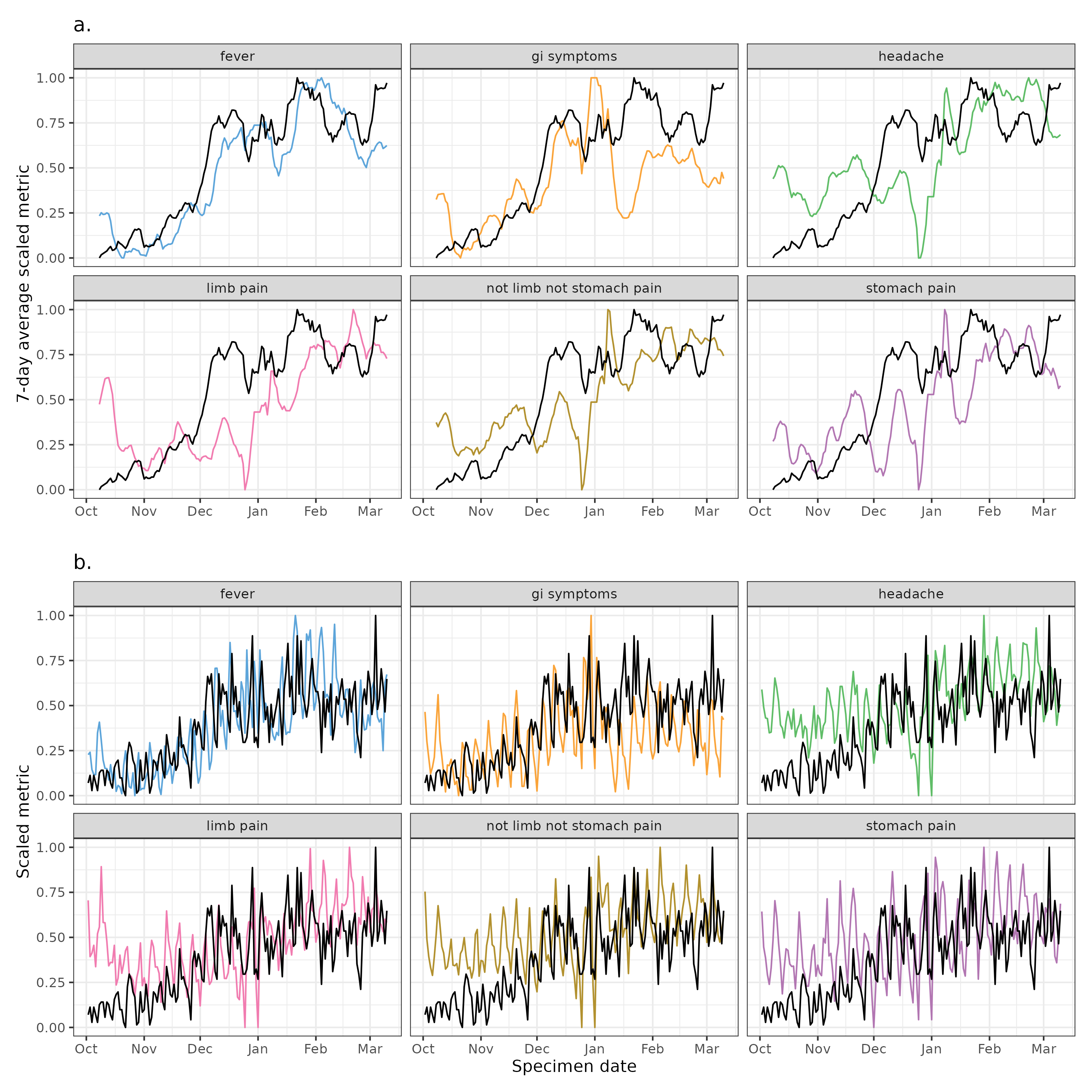

Supplement: S2 Fig — The signals are scaled between 0-1, a.) shows the rolling 7-day mean values for indicator and case trend, where b.) shows the unsmoothed more stochastic data with day-of-week effects. (TIF) [file pcbi.1012849.s006.tif]

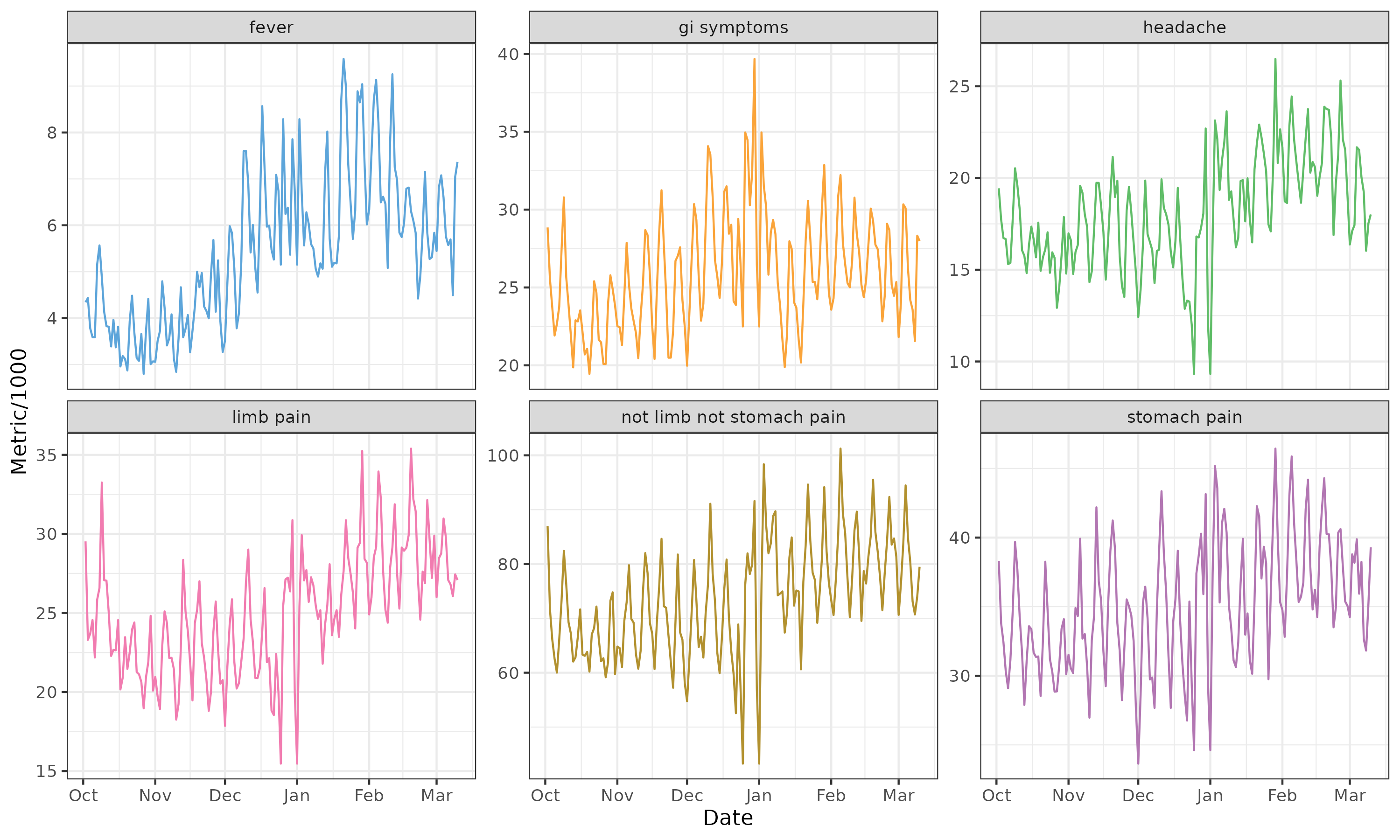

Supplement: S3 Fig — The more generic symptom categorisations such as “all pain” have larger magnitudes compared to more severe and specific symptoms such as “fever”. (TIF) [file pcbi.1012849.s007.tif]

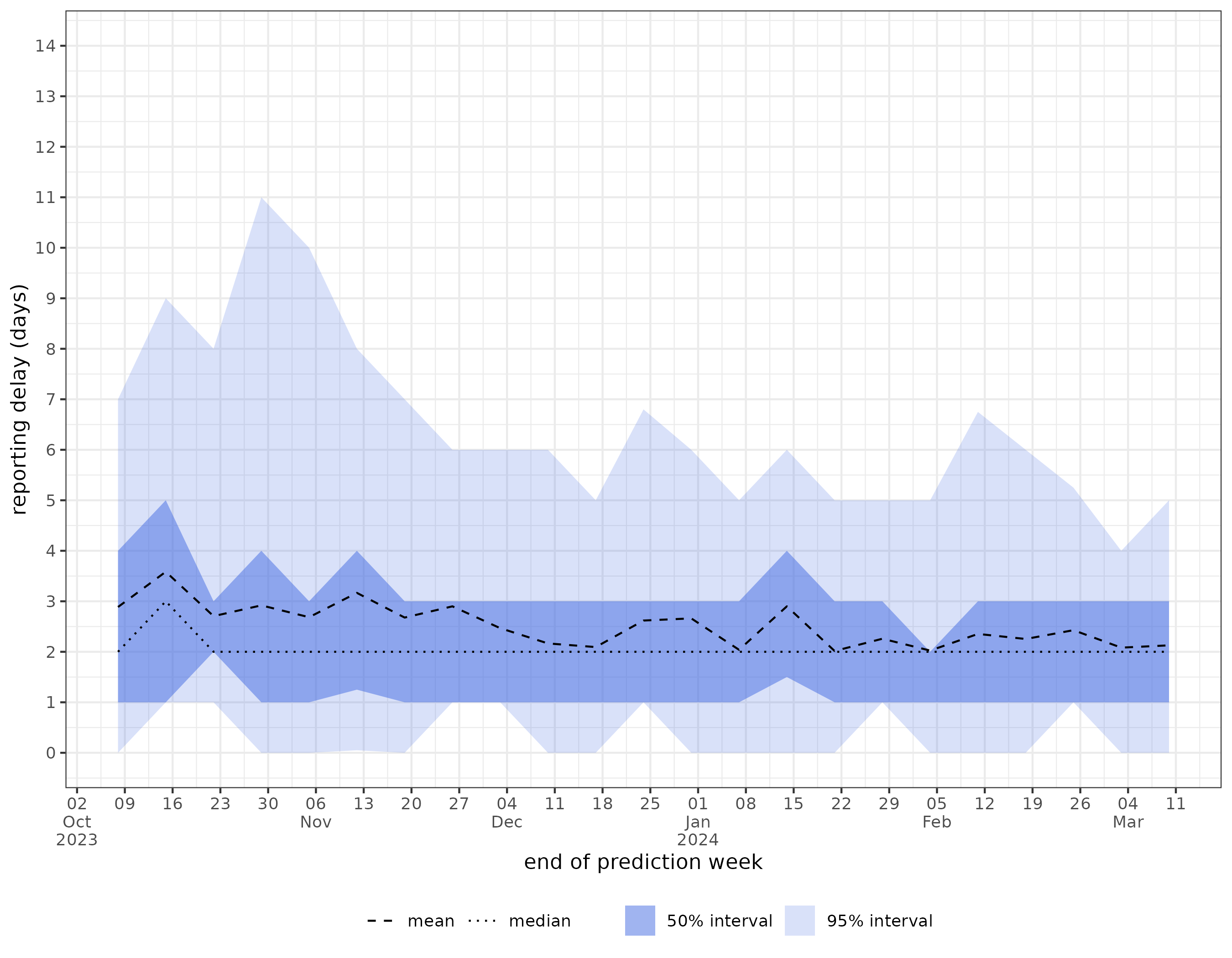

Supplement: S4 Fig — The mean, median, 95% and 50% quantile intervals are given for the time delay giving a trend over time. There is a larger tail in reporting delay early in the time series, thought this is the time with fewest positive tests. (TIF) [file pcbi.1012849.s008.tif]

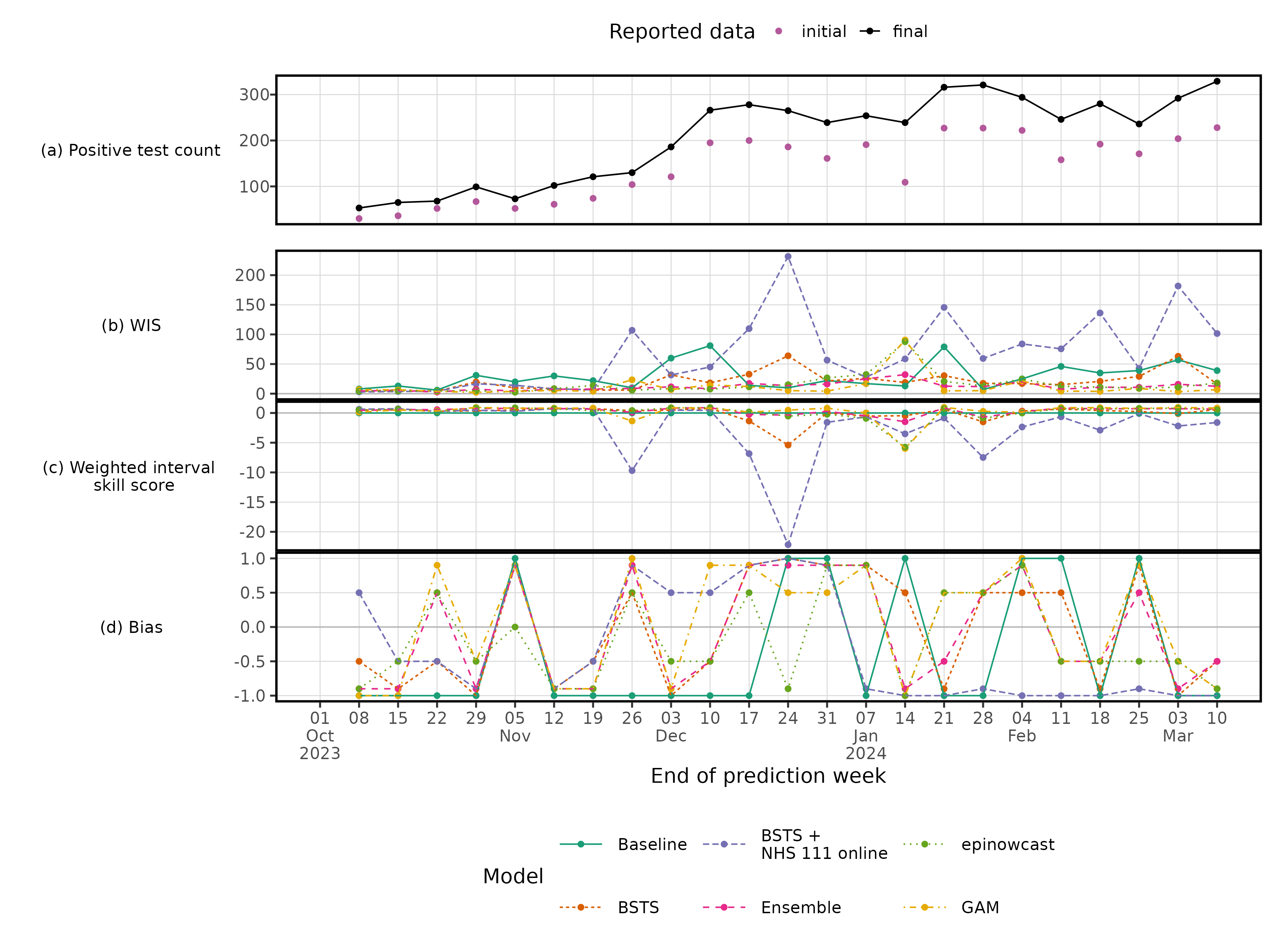

Supplement: S5 Fig — (TIF) [file pcbi.1012849.s009.tif]

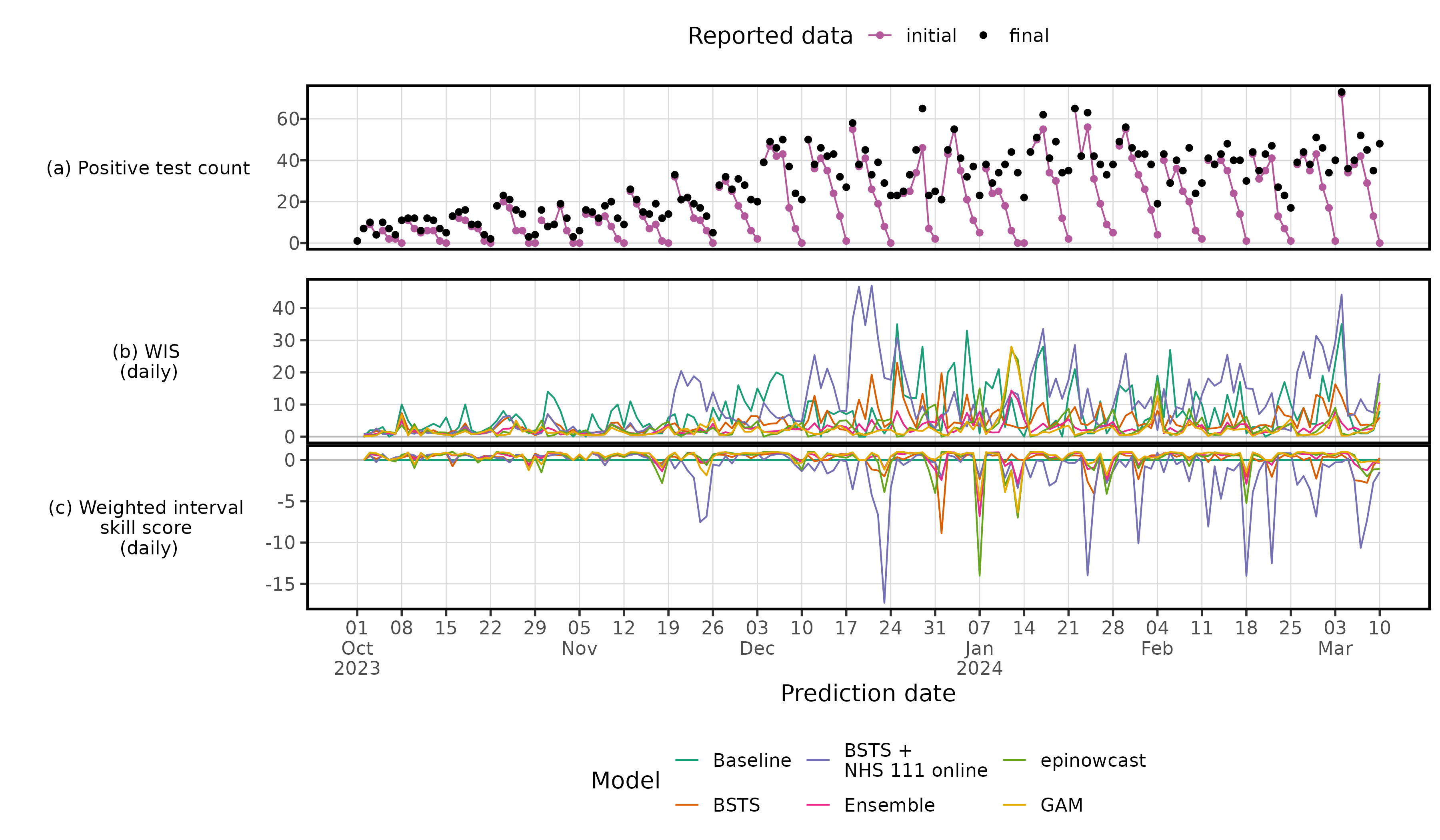

Supplement: S6 Fig — (TIF) [file pcbi.1012849.s010.tif]
